# Chemical and Region Equilibria with Heterogeneous Fluids Using Classical Density Functional Theory

**DOI:** 10.1021/acs.jpcb.5c03800

**Published:** 2025-10-27

**Authors:** Igor P. S. Pereira, Iuri S. V. Segtovich, Marcelo Castier, Henrique P. Pacheco, Frederico W. Tavares

**Affiliations:** † Programa de Engenharia Química, COPPE, 28125 Universidade Federal do Rio de Janeiro, Rio de Janeiro, RJ 21941-909, Brazil; ‡ Center for the Advancement of Technology in Society and Industry, Polytechnic University Taiwan-Paraguay, Asunción 001018, Paraguay; § Chemical Engineering Program, 146313 Texas A&M University at Qatar, Education City, Doha PO Box 23874, Qatar; ∥ Engenharia de Processos Químicos e Bioquímicos (EPQB), Escola de Química, 28125 Universidade Federal do Rio de Janeiro, Rio de Janeiro, RJ 21941-909, Brazil

## Abstract

Classical density functional theory has played an important role in adsorption calculations. However, until now, this tool has not been used for adsorption calculations in reactive systems. In this work, a formulation is proposed for minimizing the Helmholtz energy of systems formed by regions of homogeneous and heterogeneous fluids, which can participate in multiple reversible chemical reactions. This methodology allows the determination of not only the saturation conditions (adsorption isotherms) but also of the molar partition of the components between the system’s regions. In particular, the results show how external potentials of heterogeneous fluids can influence the overall conversion in reactive systems.

## Graphical abstract

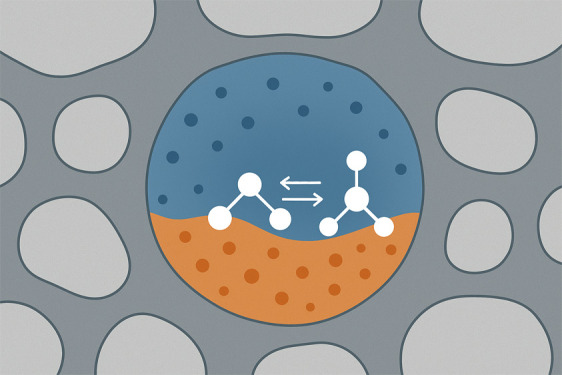

## 1 Introduction

The determination of the thermodynamic equilibrium condition of reactive multiphase systems has been the subject of many publications over the years in the field known as Chemical and Phase Equilibrium (CPE) calculations. Various authors have contributed to developing algorithms, most notably by using Gibbs energy minimization (GEM). In the Tsanas et al.^1,2^ articles, the authors made a detailed review of the historical evolution through which GEM methods have undergone.

The growing demand for research into Carbon Capture, Storage, and Utilization (CCUS) is expanding the interest in CPE, as the geological mineralization of CO_2_ is proving to be a promising storage technique. In this context, a series of chemical reactions between ions from the speciation of CO_2_ and rock minerals leads to the precipitation of carbonates, which are insoluble in the aqueous phase. The work by Leal et al.^3–5^ addresses this type of problem using a GEM technique developed by the authors and implemented in Reaktoro,^6^ a general-purpose software that is widely used for CPE problems in geological reservoirs. Besides that, as indicated by Leal et al.,^5^ the GEM technique is the basis of many reference software packages for equilibrium calculations in systems of geological interest, which consist of various solid mineral phases, as well as electrolytic aqueous solutions, gases, and supercritical fluids.

Moreover, GEM techniques are also applied to help with the assessment of systems with heterogeneous reactions. Such fluid–solid interactions could pose as a challenge given the often convoluted electronic interactions between reactants, intermediates and catalysts. Nonetheless, GEM offers a way to predict equilibrium states, which is particularly useful for systems with parallel reactions. Usually, the strategy is to apply GEM to the reaction with and without a catalyst. The presence of a catalyst can activate reactions that typically have very slow kinetics. Thus, additional constraints for Gibbs minimization need to be considered depending on the presence of the catalyst. Therefore, the role of the catalyst in calculating the chemical equilibrium condition is to select which chemical reactions should or should not take place in the system. By observing the differences between these two setups, it is possible to further understand how the catalyst influences the overall reaction mechanism and selectivity. This approach has been employed in several reacting systems, including water–gas shift reaction, CO methanation,^7^ CO_2_ upgrading to methanol and to dimethyl ether,^8^ and catalytic pyrolysis.^9^ For instance, analyzing the latter case, the authors took advantage of GEM to predict the product distributions of olefins at several operating conditions (temperature, pressure, catalyst), which could then be confirmed through experimentation.

However, the conventional techniques used in CPE are not capable of dealing with the heterogeneities that arise when fluids are adsorbed in the pores of rocks because they are based on the assumption that each phase present is homogeneous. In the work by Dawass et al.,^10^ the equilibrium condition of nonreactive systems with heterogeneous regions was obtained using equations of state for confined fluids (CF-EOS),^11–17^ which can be understood as modifications of equations of state (EOS) for describing the adsorption phenomenon, but which have limited application and accuracy. The behavior of heterogeneous fluids has received much attention from the adsorption community,^18–27^ which has started to use Classical Density Functional Theory (cDFT)^28–30^ as a viable alternative to Grand-Canonical Monte Carlo (GCMC) simulations, preserving accuracy and drastically reducing computational costs.^26,27,31^ However, until now, this technique has not been used for reactive systems, and most of its applications have been in the grand-canonical *ensemble*. In the papers of Hernando^32^ and Lutsko,^33,34^ the structure of the cDFT is recreated for the canonical *ensemble* and compared with that of the grand-canonical *ensemble*. In this context, the authors incorporated the specifications of the number of particles by means of Lagrange multipliers in the grand canonical potential. However, the possibility of integrating multiple regions into the system has not been explored. Importantly, González et al.,^35^ Kosov et al.,^36^ de las Heras and Schmidt^37^ discussed the equivalence between equilibrium density distributions in the canonical and grand-canonical *ensembles*, showing that differences arise only in small systems (*N* ≲ 10^2^).

In this work, inspired by the work of Dawass et al.,^10^ a theoretical and computational formulation is presented for minimizing the Helmholtz free energy (HEM) of reactive systems with heterogeneous regions, whose modeling can be done by means of cDFT free energy functionals. A computational example is discussed exploring the effect that confinement can have on the system’s equilibrium condition with reactions. The example discussed here serves as proof of concept for the formulation. Even with its simplicity, it already shows the effects of external potential on the equilibrium condition. That said, our main objective is to present the formulation and indicate some possible applications.

## 2 Methodology

This section focuses on describing the formulation through which the equilibrium condition of a reactive system with homogeneous and heterogeneous regions will be obtained. The strategy consists of minimizing the Helmholtz free energy of the system while keeping the temperature, the volumes of the two regions, and the abundance of the elements constant. The latter constraint is imposed by means of Lagrange multipliers. We will begin by presenting the formulation of the problem itself. Next, we will provide a microscopic description of the heterogeneous region. Then, we will show how the Helmholtz free energies of each region are calculated and how to integrate information from the microscopic scale with the macroscopic scale. Subsequently, we will briefly discuss how to insert the properties of the reference states into the formulation. Finally, we will suggest a numerical method for solving the problem with the appropriate algorithm.

### 2.1 Chemical and Phase Equilibria Calculations

Suppose a system with *N*_*c*_ components composed of *N*_*e*_ elements in which reversible chemical reactions take place. A closed rigid isothermal system can be described by means of volume (*V*), temperature (*T*), and the total number of atoms/molecules of each element (***b***) that are conserved. The equilibrium condition of this system – a *bV T ensemble* – can be obtained by

$$\min\;\beta F_{t}\;\mathrm{subject}\;\mathrm{to}\;\sum_{i=1}^{N_{c}}A_{ji}n_{i}=b_{j},\forall j=1,2,\cdots ,N_{e}$$ 1

where the constraint represents the atomic or element balance. The symbol β*F*_t_ is the dimensionless total Helmholtz free energy of the system, with β = (*k*_B_ *T*)^−1^, where *k*_B_ is the Boltzmann constant and *T* is the absolute temperature; *A*_*ji*_ is the number of atoms of element *j* in a molecule of component *i*; *n*_*i*_ is the number of molecules of component *i* in the whole system; and *b*_*j*_ is the number of atoms of element *j* in the system. The central idea of a chemical equilibrium problem in the *bV T ensemble* is to determine the quantities *n*_*i*_ that minimize β*F*_t_ subject to atomic or element balance. This formulation of chemical equilibrium is commonly known as nonstoichiometric because it does not require information about the stoichiometry of the reactions involved (more specifically, the stoichiometric coefficients). However, it can be shown that this formulation and the stoichiometric formulation are equivalent and can be interchanged. In Smith and Missen,^38^ the reader can obtain more details about each of the two formulations.

If it is necessary to partition the system into *N*_*r*_ disjoint regions, the total free energy of the system β*F*_t_ is simply given by the sum of the free energies β*F*_r_ of each region. In turn, the total number of *i* molecules is the sum of the number of *i* molecules in each of the regions, i.e.

$$\min\;\sum_{r=1}^{N_{r}}\beta F_{r}\;\mathrm{subject}\;\mathrm{to}\;\sum_{i=1}^{N_{c}}A_{ji}\left(\sum_{r=1}^{N_{r}}n_{ir}\right)=b_{j},\forall j=1,2,\cdots ,N_{e}$$ 2

where *n*_*ir*_ is the number of molecules of component *i* in region *r*. In particular, considering that the system is composed of only two regions, one homogeneous (*b*), which is the bulk, and one heterogeneous (*h*), then it is therefore possible to rewrite eq 2 as

$$\min(\beta F_{b}+\beta F_{h})\;\mathrm{subject}\;\mathrm{to}\sum_{i=1}^{N_{c}}A_{ji}(n_{i}^{b}+n_{i}^{h})=b_{j},\forall j=1,2,\cdots ,N_{e}$$ 3

where β*F*_b_ and β*F*_h_ are, respectively, the dimensionless Helmholtz free energies of the bulk and of the heterogeneous fluid, which will be described in a later subsection; *n*_*i*_^b^ and *n*_*i*_^h^ are the number of molecules of component *i* in the bulk and in the heterogeneous fluid, respectively.

### 2.2 Density Distribution and Scale Integration

Typically, heterogeneities manifest themselves on a microscopic scale despite the domain H possibly being, on a macroscopic scale, of the same order of magnitude as the scale of the system. All partitions of the system—including heterogeneous regions and the bulk phase—can be modeled using cDFT, by conceptualizing the bulk as being confined within a large, well-defined geometrical reservoir, and describing the external field in the whole domain. This approach yields highly accurate results but involves significant formulation complexity and considerable computational cost. Alternatively one can choose a microregion size that can be feasibly be simulated with cDFT, and whose properties can somehow be related to the macroregion, such a strategy to integrate scales will be discussed next. In order to connect the scale at which heterogeneity effects occur with the macroscopic scale, imagine that the heterogeneous region H has average macroscopic number densities {ρ_*i*_^h^}, such that ρ_*i*_^h^ = *n*_*i*_^h^/*V*_h_, where *V*_h_ is the heterogeneous region volume. However, on the microscopic scale, the average density of a component *i* in a microregion H_**ω**_ (whose scale shows heterogeneity) is given by

$$\left\langle \rho_{i}\right\rangle \left(\omega \right)≡\frac{1}{v_{\omega }}\int_{H_{\omega }}\rho_{i}\left(r,\omega \right)dr$$ 4

where ρ_*i*_(***r***, **ω**) is the number density distribution of component *i* in H_**ω**_, **ω** ∈ Ω is a vector of geometric parameters that differentiates two microregions and *v*_ω_ is the volume of microregion H_**ω**_. This density is a function that, for each point in a microregion, provides the local volumetric density of the component *i*. Thus, the integral of the density distribution in H_**ω**_ provides the number of particles of component *i* within the microregion. Figure 1 shows a schematic illustration of the connection between macroregion H and microregions H_**ω**_.

**Figure 1:**
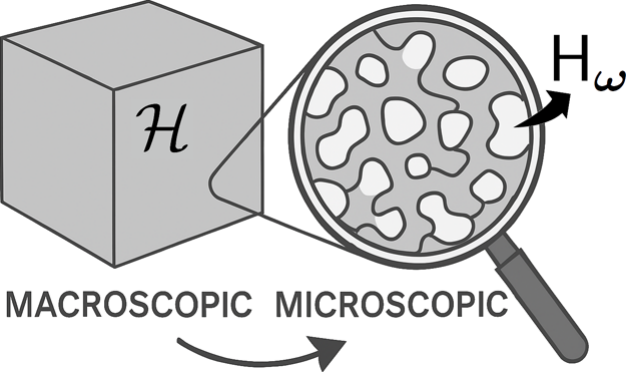
Macroscopic and microscopic view of the heterogeneous region. On the macro scale, the H region looks homogeneous, but on the micro scale, heterogeneities are evident.

Now let P(ω)dω be the fraction of the total volume occupied by microregions with parameter vector between **ω** and **ω** + d**ω**, then we can say that

$$\rho_{i}^{h}≡\frac{n_{i}^{h}}{V_{h}}=\int_{\Omega }\left\langle \rho_{i}\right\rangle \left(\omega \right)P\left(\omega \right)d\omega$$ 5

In this way, the macroscopic density of the heterogeneous region can be understood as the expected value among average microscopic densities. This applies well in many cases if we choose a suitable scale for the microscopic calculations that is larger than the length scale of the forces inducing heterogeneity, and if these forces repeat homogeneously over a larger scale (for example, uniformly mixed/dispersed adsorbent pellets where the pore distribution is the same at any point sampled of the H region). Another important point is to recognize that it is implicitly assumed that the interface between the two macroregions is being neglected in the same way that, typically, the interface between two phases is neglected in a chemical and phase equilibrium calculation for multiphase system. The contact area between the two regions contributes much less to free energy than the volume contribution of each one of them.

### 2.3 Helmholtz Free Energy

The central function for determining the equilibrium condition in the *bV T* ensemble is the Helmholtz free energy of the system. Since the system is made up of two regions, it is necessary to indicate the free energy of each of them. The free energy of the B region is a function of the temperature, volume *V*_b_, and composition ***n*** ^b^ of the bulk and can be written, omitting dependencies with the first two variables, simply as

$$\beta F_{b}\left(n^{b}\right)=\overset{N_{c}}{\underset{i=1}{\sum }}n_{i}^{b}\left[\ln\;\Lambda_{i}^{3}+\ln\left(\frac{n_{i}^{b}}{V_{b}}\right)-1\right]+\beta F_{b}^{\mathrm{exc}}\left(n^{b}\right)$$ 6

where the summation in the RHS accounts for the ideal gas contribution and the second term (*F*_b_^exc^) is the excess contribution obtained from an equation of state (EOS). The symbol Λ_*i*_ accounts for the product between the de Broglie thermal wavelength and the internal partition function of molecule *i*. It does not only include the kinetic partition function of component *i* (de Broglie thermal wavelength) but also the intramolecular degrees of freedom, such as vibrational and rotational modes, etc. This contribution is not explicitly modeled, but it will be considered in the standard chemical potential term (eq 12), which is typically tabulated in chemical reaction databases and it will be discussed in a later subsection. The partial derivatives of β*F*_b_ with respect to {*n*_*i*_^b^} correspond to the chemical potentials {βμ_*i*_^b^}, which have the following form

$$\beta \mu_{i}^{b}\left(n^{b}\right)=\ln\;\Lambda_{i}^{3}+\ln\left(\frac{n_{i}^{b}}{V_{b}}\right)+\beta \mu_{i}^{\mathrm{exc}}\left(n^{b}\right)$$ 7

The free energy functional of a heterogeneous fluid in a microregion H_**ω**_ is a generalization of eq 6 which is a functional of local number densities ρ_*i*_. If there is an external potential, it includes the explicit contribution of the energy associated with that potential as a scalar field βϕ_*i*_. Then,

$$\beta F\left[\rho ;\omega \right]=\overset{N_{c}}{\underset{i=1}{\sum }}\int_{H_{\omega }}\rho_{i}\left(r,\omega \right)\left[\ln\;\Lambda_{i}^{3}+\ln\;\rho_{i}\left(r,\omega \right)-1\right]dr+\beta F^{\mathrm{exc}}\left[\rho \right]+\overset{N_{c}}{\underset{i=1}{\sum }}\int_{H_{\omega }}\rho_{i}\left(r,\omega \right)\beta \phi_{i}\left(r,\omega \right)dr$$ 8

where the first summation in the RHS corresponds to the ideal gas contribution; the second term ( $\beta F^{\mathrm{exc}}$ ) is the excess contribution, given by a functional model consistent with bulk EOS; and the last summation is the contribution of the external potential βϕ_*i*_. The notation βF[ρ;ω] means that βF is a functional of the densities and a function of **ω**. The functional derivatives of βF play the role, for the heterogeneous fluid, of what would be the inhomogeneous chemical potentials:

$$\frac{\delta \beta F\left[\rho ;\omega \right]}{\delta \rho_{i}\left(r,\omega \right)}=\ln\;\Lambda_{i}^{3}+\ln\;\rho_{i}\left(r,\omega \right)+\frac{\delta \beta F^{\mathrm{exc}}\left[\rho \right]}{\delta \rho_{i}\left(r,\omega \right)}+\beta \phi_{i}\left(r,\omega \right)$$ 9

Now suppose that the Helmholtz free energy of the region H per unit volume, i.e. β*F*_h_/*V*_h_, is the expected value of the ratios $\beta F/v_{\omega }$ and can therefore be written as

$$\frac{\beta F_{h}}{V_{h}}=\int_{\Omega }\frac{\beta F\left[\rho ;\omega \right]}{v_{\omega }}P\left(\omega \right)d\omega$$ 10

The macroscopic Helmholtz energy depends explicitly on the macroscopic average densities, and implicitly on the microscopic average densities. Once the expression for the free energy β*F*_h_ of the macroscopic region H is known, the next step involves obtaining the form of the associated macroscopic chemical potentials {μ_*i*_^h^}, which are

$$\beta \mu_{i}^{h}=\frac{\delta \beta F[\rho ;\omega ]}{\delta \rho_{i}(r,\omega )},\forall r\in H_{\omega }\;\mathrm{and}\;\forall \omega \in \Omega$$ 11

For more details, see the Supporting Information. The equation above shows that the functional derivatives in the RHS are constant in H_**ω**_ and in Ω, i.e. they do not vary from point to point within a microregion, and their value is the same for all microregions. A consequence of this statement is that because they share open boundaries with each other, all microregions have the same heterogeneous chemical potential at equilibrium. Behind this lies the hypothesis that the free energy of one microregion does not depend on the densities of another microregion. Thus, there is a decoupling of the energies of the various microregions.

### 2.4 Reference State

Unlike a phase equilibrium calculation, a chemical equilibrium calculation requires information about the formation properties of the reference state. Thus, the reference state of component *i* is defined as a homogeneous ideal gas, pure in *i*, at the same temperature as the system and with number density ρ°, which means that its chemical potential of formation is given by (see eq 7)­

$$\beta \mu_{i}^{◦}=\ln\;\Lambda_{i}^{3}+\ln\;\rho^{◦}$$ 12

allowing to rewrite eqs 7 and 9 by eliminating the term lnΛ_*i*_^3^ in favor of the properties of the reference state, which results in

$$\beta \mu_{i}^{b}\left(n^{b}\right)=\beta \mu_{i}^{◦}-\ln\;\rho^{◦}+\ln\left(\frac{n_{i}^{b}}{V_{b}}\right)+\beta \mu_{i}^{\mathrm{exc}}\left(n^{b}\right)$$ 13

$$\frac{\delta \beta F\left[\rho ;\omega \right]}{\delta \rho_{i}\left(r,\omega \right)}=\beta \mu_{i}^{◦}-\ln\;\rho^{◦}+\ln\;\rho_{i}\left(r,\omega \right)+\frac{\delta \beta F^{\mathrm{exc}}\left[\rho \right]}{\delta \rho_{i}\left(r,\omega \right)}+\beta \phi_{i}\left(r,\omega \right)$$ 14

The values of μ_*i*_^°^ can be taken from tables with the formation properties of each component. This information is related to the free energy change of the reactions involved and, therefore, to the equilibrium constants of the reactions.

### 2.5 Lagrangian and Karush–Kuhn–Tucker (KKT) Equations

The first step here is to write the Lagrangian associated with the optimization problem stated in eq 3 whose primal variables are ${\left(n^{b},n^{h}\right)}^{T}\in R_{+}^{N_{c}}\times R_{+}^{N_{c}}$ and the dual variables (Lagrange multipliers) are $\lambda \in R^{N_{e}}$ . Thus, the Lagrangian *L* is a function of ***n*** ^b^, ***n*** ^h^ and **λ**, such that

$$L\left(n^{b},n^{h},\lambda \right)=\beta F_{b}\left(n^{b}\right)+\beta F_{h}\left(n^{h}\right)-\overset{N_{e}}{\underset{j=1}{\sum }}\lambda_{j}\left[\overset{N_{c}}{\underset{i=1}{\sum }}A_{ji}\left(n_{i}^{b}+n_{i}^{h}\right)-b_{j}\right]$$ 15

The associated KKT equations are obtained by doing the Lagrangian partial derivatives equal to zero, which results in the following system of equations

$$\beta \mu_{i}^{b}\left(n^{b}\right)-\overset{N_{e}}{\underset{j=1}{\sum }}\lambda_{j}A_{ji}=0,\forall i=1,2,\cdots ,N_{c}$$ 16

$$\beta \mu_{i}^{h}\left(n^{h}\right)-\overset{N_{e}}{\underset{j=1}{\sum }}\lambda_{j}A_{ji}=0,\forall i=1,2,\cdots ,N_{c}$$ 17

$$\overset{N_{c}}{\underset{i=1}{\sum }}A_{ji}\left(n_{i}^{b}+n_{i}^{h}\right)-b_{j}=0,\forall j=1,2,\cdots ,N_{e}$$ 18

The eqs 16 and 17 reveal that the H region, from a macroscopic point of view, can be seen as a homogeneous region, since its equation is similar in form to that of the bulk B region. Both regions share open boundaries and, therefore, equalizing the LHS, the equilibrium should be βμ_*i*_^h^ = βμ_*i*_^b^, which refers to the equilibrium between two homogeneous phases. The apparent simplicity of the system of equations hides the complex structure of βμ_*i*_^h^(***n*** ^h^) due to microscopic modeling. Although this system may suggest that ***n*** ^h^ are independent variables, they are actually calculated from the microscopic distributions ρ_*i*_(***r***, **ω**).

### 2.6 Numerical Method

The structure of the KKT equations indicates that, for a given **λ**, the bulk and heterogeneous fluid equations are completely decoupled. This feature suggests that the atomic or element balance equations could conveniently be solved in an outer loop, in which, in each iteration, the bulk and heterogeneous region equations are solved sequentially. As will become clear below, another advantage of this approach is the convenience of using any equation of state and GC-cDFT (Grand-canonical Classical Density Functional Theory) framework desired. Figure 2 shows a schematic of the Helmholtz energy minimization algorithm proposed in this work. After initializing the input variables, the bulk equation is solved for an initial estimate of **λ** and, then, the heterogeneous fluid equation is solved sequentially. It then checks whether the atomic balance is satisfied: if it is not, it updates the Lagrange multipliers λ_*j*_ using some method (e.g., Newton) and returns to the step of solving the bulk equation; if it is, the algorithm is terminated by returning the quantities of each component in the two regions. The convergence check can be done by evaluating whether ∥ ***A*** (***n*** ^b^ + ***n*** ^h^) – ***b*** ∥_2_ < ε.

**Figure 2:**
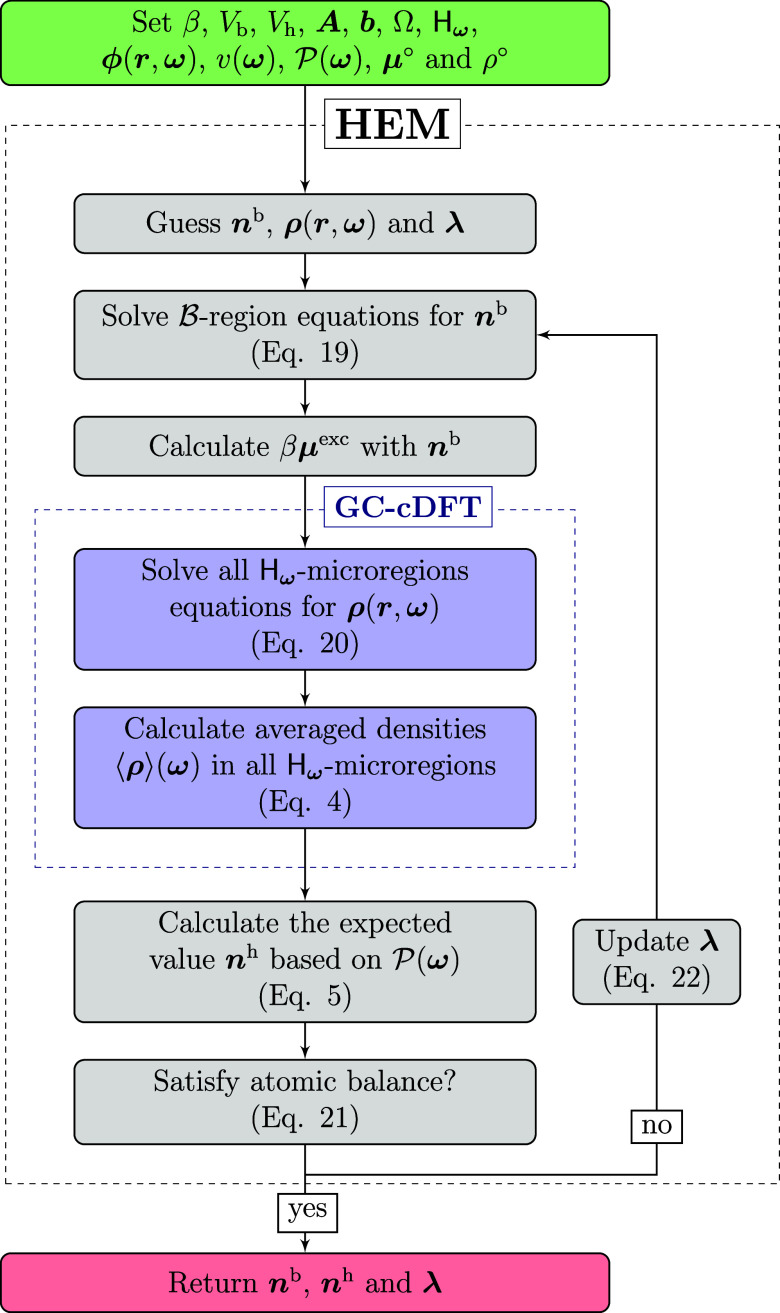
Helmholtz energy minimization algorithm flowchart. The algorithm starts by defining the input variables for the system and the model. Next, initial estimates are given for the amounts in the bulk, the microscopic density distributions, and the Lagrange multipliers. The bulk equation is solved, the chemical potentials are calculated and fed into a GC-cDFT framework, which returns the average of the microscopic density distributions. Then, the expected value of the average densities is calculated from the probability density distribution of each microregion. It is verified whether the mass balance is satisfied. If so, the algorithm ends by returning the amounts of matter in each region. If not, it updates the Lagrange multipliers and returns to the step of solving the bulk equation.

Given this sequential logic, each iteration of the HEM first updates the *n*_*i*_^b^ and then the ρ_*i*_(***r***) distributions. Thus, for given λ_*j*_, the system of equations ( B -region equations)

$$\beta \mu_{i}^{◦}-\ln\;\rho^{◦}+\ln\left(\frac{n_{i}^{b}}{V_{b}}\right)+\beta \mu_{i}^{\mathrm{exc}}\left(n^{b}\right)-\overset{N_{e}}{\underset{j=1}{\sum }}\lambda_{j}A_{ji}=0$$ 19

is solved for *n*_*i*_^b^. The publications of Tsanas^1,2^ have tackled the resolution of such a system in the context of CPE and the details can also be found in Michelsen and Møllerup.^39^ Once the bulk solution has been obtained, the bulk chemical potential is calculated with eq 13. According to eqs 16 and 17, the macroregions B and H are in equilibrium since βμ_*i*_^h^ = βμ_*i*_^b^. Then, from eq 11, it follows that $\beta \mu_{i}^{b}=\delta \beta F\left[\rho ;\omega \right]/\delta \rho_{i}\left(r,\omega \right)$ which has the same form as the GC-DFT equation, allowing one to say that

$$\rho_{i}\left(r,\omega \right)=\frac{n_{i}^{b}}{V_{b}}\exp\left[\beta \mu_{i}^{\mathrm{exc}}\left(n^{b}\right)-\frac{\delta \beta F^{\mathrm{exc}}\left[\rho \right]}{\delta \rho_{i}\left(r,\omega \right)}-\beta \phi_{i}\left(r,\omega \right)\right]$$ 20

This allows any available GC-cDFT framework to perform this step. Several authors^40–43^ have described the numerical and computational methodology involved in solving GC-cDFT equations.

The update of the Lagrange multipliers λ_*j*_ can be done by applying a numerical method for solving a system of equations, such as the Newton–Raphson method. The system deals with the atomic balances imposed by the equations whose residuals are defined as a function of the multipliers **λ** ^*k*^ in any *k* iteration, which in matrix notation, is as follows:

$$G_{k}=G\left(\lambda^{k}\right)≡A\left[n^{b}\left(\lambda^{k}\right)+n^{h}\left(\lambda^{k}\right)\right]-b$$ 21

The update of the multipliers is given by

$$\lambda^{k+1}=\lambda^{k}-\zeta J_{k}^{-1}G_{k}$$ 22

where ζ ∈ (0, 1] is a step control parameter and $J_{k}=\nabla G\left(\lambda^{k}\right)$ is the Jacobian matrix of G , i.e.

$$J=[\frac{\partial G_{j}}{\partial \lambda_{l}}{]}_{N_{e}\times N_{e}}=\nabla G=A\left[\nabla n^{b}+\nabla n^{h}\right]$$ 23

which can be obtained via automatic differentiation, since ∇ ***n*** ^b^ and ∇ ***n*** ^h^ do not have explicit expressions, in general, due to the transcendental dependencies of ***n*** ^b^ and ***n*** ^h^ presented by βμ_*i*_^exc^ and $\frac{\delta \beta F^{\mathrm{exc}}}{\delta \rho_{i}\left(r\right)}$ , respectively. However, for the particular case of ideal gases, the bulk and heterogeneous fluid eqs (eqs 19 and 20) have an analytical solution given by

$$n_{i}^{b}=\rho^{◦}V_{b}\exp\left[-\beta \mu_{i}^{◦}+\overset{N_{e}}{\underset{j=1}{\sum }}\lambda_{j}A_{ji}\right]$$ 24

$$\rho_{i}\left(r,\omega \right)=\frac{n_{i}^{b}}{V_{b}}\exp\left[-\beta \phi_{i}\left(r,\omega \right)\right]$$ 25

which, substituting in eqs 4 and 5, results in

$$n_{i}^{h}=V_{h}\int_{\Omega }\left[\frac{1}{v\left(\omega \right)}\int_{H_{\omega }}\frac{n_{i}^{b}}{V_{b}}\exp\left[-\beta \phi_{i}\left(r,\omega \right)\right]dr\right]P\left(\omega \right)d\omega$$ 26

Note that, in this case, the Jacobian ***J*** can be explicitly written as follows:

$${\left(\nabla n^{b}\right)}_{il}=\frac{\partial n_{i}^{b}}{\partial \lambda_{l}}=A_{li}n_{i}^{b}$$ 27

$${\left(\nabla n^{h}\right)}_{il}=\frac{\partial n_{i}^{h}}{\partial \lambda_{l}}=A_{li}n_{i}^{h}$$ 28

which leads to

$$J_{jl}=\overset{N_{c}}{\underset{i=1}{\sum }}A_{ji}\left(A_{li}n_{i}^{b}+A_{li}n_{i}^{h}\right)=\overset{N_{c}}{\underset{i=1}{\sum }}A_{ji}A_{li}\left(n_{i}^{b}+n_{i}^{h}\right)$$ 29

Defining *K*_*li*_ = *A*_*li*_(*n*_*i*_^b^ + *n*_*i*_^h^), the previous equation can then be rewritten as ***J*** = ***A*** ***K*** ^*T*^. Even for nonideal systems, it is believed that the matrix ***A*** ***K*** ^*T*^ can serve as a good estimate for the Jacobian ***J***.

## 3 Results and Discussion

In this section, we present a case study that serves as proof of concept for the formulation described in the previous section. Its simplicity allows us to explore the implications of the formulation without getting bogged down in complications arising from the complexity of the model. For this reason, we chose an example of a single reaction, with the ideal gas model in a simplified pore. Other examples are presented in the Supporting Information.

### 3.1 Effect of External Potential on Chemical Conversion: A Reactive Adsorption Example

This case study has no precedent in the literature. Its goal is to demonstrate the potential of the methodology formulated in this work. Suppose that a gas comprised of 1 mol of H_2_ and 1 mol I_2_ reacts according to the following reversible reaction

$$H_{2}+I_{2}⇌2\mathrm{HI}$$ 30

and can be adsorbed by and adsorbent with a certain porosity φ. Suppose, also, that the adsorbent has a fixed mass *m*_*s*_ and density ρ_*s*_. Therefore, the heterogeneous volume *V*_h_ is the volume accessible for the fluid and can be written as

$$V_{h}=\varphi \frac{m_{s}}{\rho_{s}}$$ 31

Consequently, the volume of the inert solid is $\left(1-\varphi \right)\frac{m_{s}}{\rho_{s}}$ . Inside the pores of this adsorbent, the adsorbed gas behaves heterogeneously because of their interaction with the pore walls. Thus, the system can be partitioned into one region as a homogeneous gas in the bulk and another as a heterogeneous fluid, a gas confined/adsorbed in the pores of the adsorbent. Therefore, the objective is to analyze how confinement shifts the chemical equilibrium condition, compared to the unconfined case or, in other words, how the conversion varies when the bulk volume *V*_b_ changes due to the action of a piston. Figure 3 shows a schematic diagram of the piston system.

**Figure 3:**
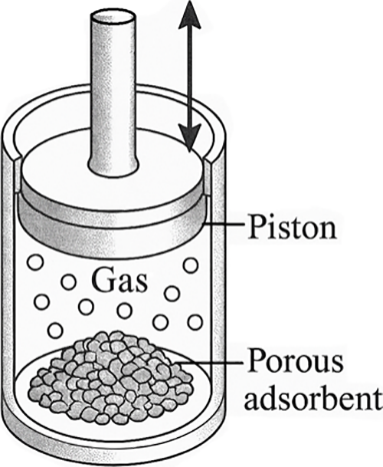
Schematic representation of the system. The reactant gases (H_2_ and I_2_) and the adsorbent material are inside the vessel. By means of a piston, the volume available for the gas in the bulk can be varied.

To do this, it will be assumed that the fluid behaves as an ideal gas in both regions and that the pores of the adsorbent material can be represented by a slit pore with width *H* and a cross-sectional area of 1 Å^2^ as shown in Figure 4. The fluid–solid interaction will be modeled using the Steele-type external potential,^44^ which, accounting for the effect both walls, takes the form

$$\beta \phi_{i}\left(z\right)=\frac{\epsilon_{i}}{T}[\frac{2}{5}(\frac{\sigma_{i}}{z+0.5H}{)}^{10}-\left(\frac{\sigma_{i}}{z+0.5H}{)}^{4}-\frac{{\sigma_{i}}^{4}}{10.05Å\cdot {\left(2.0435Å+z+0.5H\right)}^{3}}\right]+\frac{\epsilon_{i}}{T}[\frac{2}{5}(\frac{\sigma_{i}}{0.5H-z}{)}^{10}-\left(\frac{\sigma_{i}}{0.5H-z}{)}^{4}-\frac{{\sigma_{i}}^{4}}{10.05Å\cdot {\left(2.0435Å+0.5H-z\right)}^{3}}\right]$$ 32

where *z* is the spatial coordinate corresponding to the distance, measured in Å, from the center of the pore whose width is *H*; ϵ_*i*_ and σ_*i*_ parameters (see Table 1) of the *i* component in Kelvin and Å, respectively. In this case, where the heterogeneous region H is represented by slit pores, the microregions H_**ω**_ are characterized by a single geometric parameter, the pore width, and its volume is *v*(ω) = ω · 1Å^2^. Thus, the Ω space is, in this case, the interval containing all possible values for a pore width, i.e., ω ∈ [0, ∞), and the distribution P is related to that which, in the adsorption literature, is called Pore Size Distribution (PSD).^45^ Furthermore, as the adsorbent is supposed to be made up of replicas of just one pore with a width of *H*, the probability density takes the form

P(ω)=δ(ω−H) 33

where δ is the Dirac delta function. As a result, the whole region H is constructed solely by *H*_0_, with volume *v* = *H* · 1Å^2^, and all integrals of the form $\int_{0}^{\infty }\chi \left(\omega \right)P\left(\omega \right)d\omega$ can be written simply as

$$\int_{0}^{\infty }\chi \left(\omega \right)\delta \left(\omega -H\right)d\omega =\chi \left(H\right)$$ 34

where χ is any function of ω. Thus, one can rewrite eqs 5, 10 and 11 as follows

$$n_{i}^{h}=V_{h}\left\langle \rho_{i}\right\rangle \left(H\right)$$ 35

$$\beta F_{h}=\frac{V_{h}}{v}\beta F\left(H\right)$$ 36

$$\beta \mu_{i}^{h}=\frac{\delta \beta F\left(H\right)}{\delta \rho_{i}\left(r,H\right)}$$ 37

**Figure 4:**
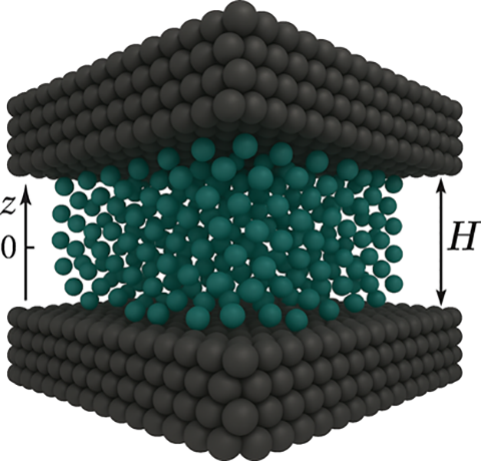
Slit pore with a width equal to *H*. The symmetry present makes it possible to model this type of pore with a one-dimensional domain (*z* axis) whose origin is located in the center of the pore. The black spheres represent the solid particles while the blue spheres represent the fluid particles. Figure merely illustrative of pore geometry, since the model used does not provide an atomistic description for either the solid or the gas.

**Table 1:** Steele Potential Parameters Values and Standard Chemical Potentials for Iodine Example Species

				βμ_*i*_^°^			
*i*	species	ϵ_*i*_ [K]	σ_*i*_ [Å]	373 K	573 K	773 K	973 K
1	H_2_	951.03	3.1135	–0.0813	–0.6075	–1.1867	–1.7190
2	I_2_	5064.97	4.2800	2.6302	–5.0821	–9.2362	–11.9395
3	HI	3124.32	3.8055	–1.5448	–5.0564	–7.0858	–8.4841

In order to analyze the effect of the external potential on the conversion of the reaction (eq 30), the total volume (*V*_h_) was set at 1 L and the conversion of H_2_ was calculated as a function of the ratio *V*_b_/*V*_h_ for various pore widths (4, 5, 7, 10, 15, 20, and 10^4^ times 2.827 Å) and different temperatures (373, 573, 773, and 973 K), at which the standard chemical potentials are reported by Table 1 and calculated from Poling et al.^46^ data. The conversion has been defined as

$$X_{i}=\frac{n_{i}^{f}-n_{i}^{b}-n_{i}^{h}}{n_{i}^{f}},\mathrm{for}\;a\;\mathrm{reactant}\;i$$ 38

where *n*_*i*_^f^ is the number of added moles of component *i*, i.e. ***n*** ^f^ = [1, 1, 0]^*T*^ mol such that ***b*** = ***A*** ***n*** ^f^, where

$$A=[\begin{array}{ccc} 2 & 0 & 1\\ 0 & 2 & 1 \end{array}]$$

Since the amounts added of the two reagents (H_2_ and I_2_) are the same and the stoichiometry of the reaction (eq 30) obeys H_2_:I_2_ = 1, then the conversion of these components is equal and will be denoted simply by *X*.

The first effect that can be observed is the reduction in bulk pressure *P*_b_ with the increase in bulk volume *V*_b_ (first column of Figure 5). For small volumes of bulk, the pressure does not diverge because the action of adsorption also causes *n*_*i*_^b^ → 0. Lower temperatures favor adsorption as the magnitude of the external potential increases (see eq 32). Similarly, systems formed by smaller pores also have greater adsorptive capacity since the action of the external potential near the walls is manifested throughout the entire volume *v* of the microregion. On the other hand, large pores have a large portion of their volume in which the external potential is nearly zero and, therefore, does not contribute as much to adsorption. It can be observed that systems with smaller pores at lower temperatures are so favorable to adsorption that the bulk pressure does not seem to vary with an increase in bulk volume. In other words, a reduction in bulk pressure does not lead to desorption in the same way in systems with large pores and/or at high temperatures. Of course, in practice, this limit cannot be exploited as much due to impediments associated with the volume excluded at high confinement, which ideal gas molecules do not experience. The other limiting behavior is illustrated by the black curve in which the pore is so large that the portion of *v* affected by the external potential is practically negligible, and, consequently, the entire heterogeneous region behaves virtually as an extension of the bulk phase. For this scenario, in particular, the bulk pressure varies as the system can almost be seen as a single bulk phase. For this reason, from now on, the system composed of such pores will be used as a reference for a fully bulk system. Note that with *V*_b_/*V*_h_ →∞, the total available pore volume is approximately zero regardless of pore size. Therefore, all 7 systems behave as bulk only. At this point, where *V*_h_ ≪ *V*_b_, is the only configuration that satisfies the hypothesis that the bulk behaves as a particle reservoir for the heterogeneous fluid inside the pores.

**Figure 5:**
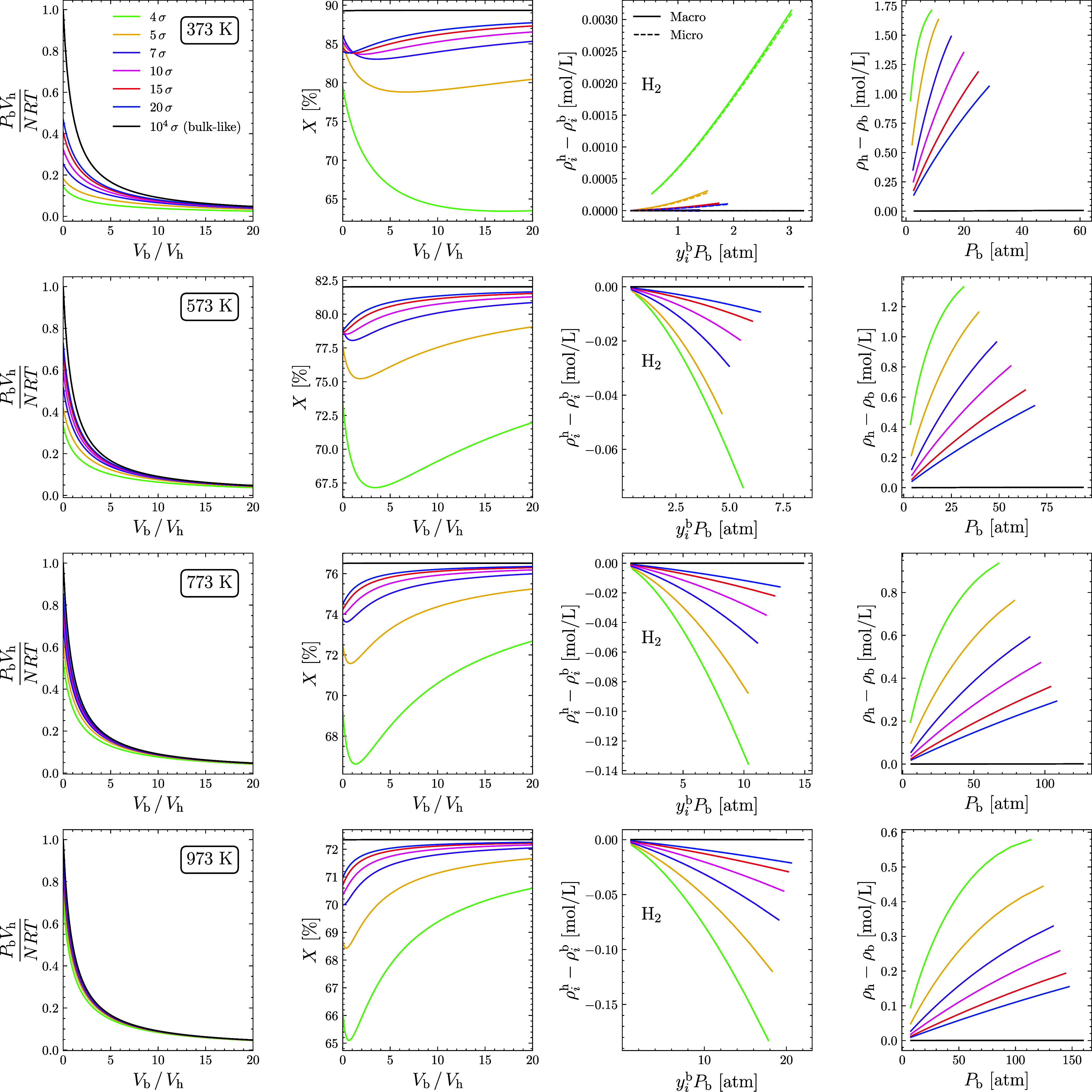
Equilibrium conditions for the system formed by H_2_, I_2_, and HI. In the first column, the bulk pressure as a function of the ratio *V*_b_/*V*_h_. In the second column, the overall/global (in bulk and adsorbed) conversion as a function of the *V*_b_/*V*_h_ ratio. In the third column, the excess adsorption isotherm per volume for hydrogen (*i*) as a function of its partial pressure in bulk. In the fourth and last column, excess isotherm per volume of the gas mixture as a function of bulk pressure. Each line is associated with a different temperature: 373, 573, 773, and 973 K respectively. Curves correspond to systems with pore sizes equal to 4 (green), 5 (yellow), 7 (purple), 10 (pink), 15 (red), 20 (blue), and 10^4^ (black) times 2.827 Å.

With regard to conversion (second column of Figure 5), it can be seen that for all 7 systems at all 4 temperatures, gas adsorption leads to a reduction in conversion compared to the fully bulk system. Furthermore, all the curves show a minimum conversion. This indicates that a given *V*_b_/*V*_h_ value leads to the lowest possible overall conversion, and this minimum is lower the smaller the pore size. It is reasonable that this minimum point should be higher in systems with larger pore sizes. After all, at the limit where *H* →∞, the conversion has to return to its fully bulk value. As the analyzed reaction preserves the total number of moles *N* = ∑ _*i* = 1_^*N*_*c*_^ *n*_*i*_^f^, the bulk pressure, although it can vary, has no influence on the equilibrium condition. Thus, the decline in conversion compared to the fully bulk system is due to the action of the external potential, which promotes adsorption. So, the apparent influence of pressure on the equilibrium condition is not actually a direct manifestation. As the volume of the bulk becomes larger and larger compared to the volume of the heterogeneous region, all other 6 systems tend asymptotically toward the conversion of the fully bulk system (black curve) as expected. At *V*_b_/*V*_h_ = 0, the system has no volume in the bulk and therefore all the gas is adsorbed and presents a certain conversion. As the piston provides the system with volume in the bulk phase, the pressure begins to decrease and, initially, H_2_ tends to desorb preferentially (the lowest value of ϵ among the three species, as shown in Table 1). Since, at this point in the diagram, the pore volume still dominates the total volume of the system, the overall conversion (especially in the pores) decreases due to this migration of H_2_ to the bulk. Conversion then reaches a minimum point. From this point on, the volume of the bulk dominates the volume of the system and the pressure has dropped enough to significantly desorb the other species. Now, conversion (especially in the bulk) increases again as the reactants tend to meet back up in the bulk. At higher temperatures, adsorption is weakened, and all systems tend to resemble the fully bulk system with increasingly smaller *V*_b_/*V*_h_ ratios.

Finally, suppose the average density in the heterogeneous macroregion and the bulk pressure can be calculated for each configuration of the system. In this case, it is possible to construct volumetric adsorption isotherms (third and fourth columns of Figure 5) that relate these two quantities. In particular, in the third column, the density of H_2_ in the heterogeneous region is calculated in two ways: i. using *n*_*i*_^h^/*V*_h_ (Macro); and ii. using eq 4 (Micro). The agreement between the two methods of calculation confirms the validity of eq 35 and illustrates that it is possible to construct the isotherm with information from either of the two scales. The fourth and final column shows the adsorption isotherm of the fluid as a whole where ρ_r_ = ∑ _*i* = 1_^*N*_*c*_^ρ_*i*_^r^, where *r* ∈{h, b} is one of the two regions.

## 4 Conclusions

A theoretical and computational formulation for minimizing the Helmholtz free energy subject to elementary balances was presented. This procedure makes it possible to calculate the equilibrium condition in ensemble *bV T* for a system made up of regions of homogeneous and heterogeneous fluids, which can take part in reversible chemical reactions. In this formulation, the heterogeneous region is modeled by means of a Helmholtz free energy functional and external potential typical of the Classical Density Functional Theory framework. In turn, the homogeneous phase must be treated with the equation of state consistent with the heterogeneous fluid model.

In order to explore the ability to deal with heterogeneous fluid-containing systems, an illustrative example of an adsorption system in which a chemical reaction takes place was used. A gas being adsorbed by a material inside which the gas is confined in pores behaves as a heterogeneous fluid. This shows the effect of the external potential on the conversion of the system. This effect could not be noticed if the chemical equilibrium condition were obtained only for the bulk. In the case of the solved example, the conversion reaches a minimum as the pressure in the bulk is varied. Thus, for a given material, there is a pressure that minimizes conversion even though the pressure does not have a direct influence on the equilibrium due to Le Chatelier’s principle, but rather an indirect influence on adsorption.

The formulation creates possibilities for more advanced applications in adsorption. In this sense, pores with three-dimensional geometry and/or pore size distribution, and nonideal fluids modeled by more sophisticated functionals can be explored. With this, systems relating to CO_2_ mineralization, for example, can have their equilibrium conditions studied on multiple scales.

## Supplementary Material



## Notes

Deducing the coarse chemical potential of the heterogeneous macroregion and other case studies (PDF)
