# Supplementary material for: Chemical and Region Equilibria with Heterogeneous Fluids Using Classical Density Functional Theory
Source: J Phys Chem B. 2025 Oct 27;129(44):11564–73. doi: 10.1021/acs.jpcb.5c03800 (PMC12598872; doi:10.1021/acs.jpcb.5c03800)
Supplement: Supplementary file 1 [file jp5c03800_si_001.pdf]

Supporting Information: *Chemical and Region Equilibria with Heterogeneous Fluids Using Classical Density Functional Theory*

Igor P. S. Pereira,[†] Iuri S. V. Segtovich,[†] Marcelo Castier,[‡,¶] Henrique Poltronieri Pacheco,[†] and Frederico W. Tavares[*,†,§]

†*Programa de Engenharia Química, COPPE, Universidade Federal do Rio de Janeiro, Rio de Janeiro, RJ 21941-909, Brazil*

‡*Center for the Advancement of Technology in Society and Industry, Polytechnic University Taiwan-Paraguay, Asunción 001018, Paraguay*

¶*Chemical Engineering Program, Texas A&M University at Qatar, Education City, PO Box 23874, Doha, Qatar*

§*Engenharia de Processos Químicos e Bioquímicos (EPQB), Escola de Química, Universidade Federal do Rio de Janeiro, Rio de Janeiro, RJ 21941-909, Brazil*

E-mail: tavares@eq.ufrj.br

Deducing the coarse chemical potential of the heterogeneous macroregion

In this section, we will demonstrate the connection between the local chemical potentials of the micro-regions and the coarse chemical potential of the heterogeneous macro-region.

Recall that

$$\langle\rho_i\rangle(\boldsymbol{\omega}) = \frac{1}{v_{\boldsymbol{\omega}}}\int_{\mathsf{H}_{\boldsymbol{\omega}}} \rho_i(\boldsymbol{r},\boldsymbol{\omega})\,\mathrm{d}\boldsymbol{r}\,, \tag{S1}$$

is the average number density within the micro-region $\mathsf{H}_{\boldsymbol{\omega}}$,

$$\rho_i^{\mathrm{h}} \equiv \frac{n_i^{\mathrm{h}}}{V_{\mathrm{h}}} = \int_\Omega \langle\rho_i\rangle(\boldsymbol{\omega})\,\mathcal{P}(\boldsymbol{\omega})\,\mathrm{d}\boldsymbol{\omega} \tag{S2}$$

and

$$\frac{\beta F_{\mathrm{h}}}{V_{\mathrm{h}}} = \int_\Omega \frac{\beta\mathcal{F}[\boldsymbol{\rho};\boldsymbol{\omega}]}{v_{\boldsymbol{\omega}}}\,\mathcal{P}(\boldsymbol{\omega})\,\mathrm{d}\boldsymbol{\omega} \tag{S3}$$

are respectively the macroscopic number density and Helmholtz energy density of the heterogeneous region. Then, the procedure for calculating macroscopic chemical potential is as follows: imagine that βF_{h} can be interpreted as functionals of $\rho_i(\boldsymbol{r},\boldsymbol{\omega})$ implicitly (see Eq. S3). As an average macroscopic property, βF_{h} should explicitly depend only on the average macroscopic densities ρ_i^{h}. These, in turn, according to Eq. S2, depend on the microscopic average densities $\langle\rho_i\rangle(\boldsymbol{\omega})$, which explicitly depend on the microscopic density distributions $\rho_i(\boldsymbol{r},\boldsymbol{\omega})$ by Eq. S1. Thus, the functional derivatives of βF_{h} in relation to the microscopic distributions $\rho_i(\boldsymbol{r},\boldsymbol{\omega})$ can be obtained by applying the chain rule. Note that the macroscopic density of a component does not depend on the microscopic densities of the other components (see Eq. S1 and S2). Therefore, the application of the chain rule for functional derivatives[1] can be simplified to obtain

$$\frac{\delta\beta F_{\mathrm{h}}}{\delta\rho_i(\boldsymbol{r},\boldsymbol{\omega})} = \frac{\partial\beta F_{\mathrm{h}}}{\partial\rho_i^{\mathrm{h}}}\;\underbrace{\frac{\delta\rho_i^{\mathrm{h}}}{\delta\langle\rho_i\rangle(\boldsymbol{\omega})}}_{\mathcal{P}(\boldsymbol{\omega})}\;\underbrace{\frac{\delta\langle\rho_i\rangle(\boldsymbol{\omega})}{\delta\rho_i(\boldsymbol{r},\boldsymbol{\omega})}}_{\frac{1}{v_{\boldsymbol{\omega}}}} = \frac{\mathcal{P}(\boldsymbol{\omega})}{v_{\boldsymbol{\omega}}}\frac{\partial\beta F_{\mathrm{h}}}{\partial\rho_i^{\mathrm{h}}}\,. \tag{S4}$$

In fact, this is not the only possible way to calculate the functional derivatives in question. There is also the option of using Eq. S3, imagining that βF_{h} is an explicit functional of $\beta\mathcal{F}[\boldsymbol{\rho};\boldsymbol{\omega}]$, which in turn is a functional of all $\rho_i(\boldsymbol{r},\boldsymbol{\omega})$. Considering that the free energy of

a microregion depends only on the densities of that region and not on the others, the chain rule is

$$\frac{\delta\beta F_{\rm h}}{\delta\rho_i(\boldsymbol{r},\boldsymbol{\omega})} = \underbrace{\frac{\delta\beta F_{\rm h}}{\delta\beta\mathcal{F}[\boldsymbol{\rho};\boldsymbol{\omega}]}}_{\displaystyle \downarrow \atop \displaystyle V_{\rm h}\frac{\mathcal{P}(\boldsymbol{\omega})}{v_{\boldsymbol{\omega}}}} \frac{\delta\beta\mathcal{F}[\boldsymbol{\rho};\boldsymbol{\omega}]}{\delta\rho_i(\boldsymbol{r},\boldsymbol{\omega})} = V_{\rm h}\frac{\mathcal{P}(\boldsymbol{\omega})}{v_{\boldsymbol{\omega}}}\frac{\delta\beta\mathcal{F}[\boldsymbol{\rho};\boldsymbol{\omega}]}{\delta\rho_i(\boldsymbol{r},\boldsymbol{\omega})}\,. \tag{S5}$$

This energy decoupling of microregions introduces an approximation to the formulation. Otherwise, the chain rule would present cross terms that would account for the variation in the free energy functional of a microregion due to the variation in the densities of all other microregions. This kind of approximation is analogous to what would be a local density approximation. Equating the RHS of Eq. S4 and S5, gives

$$\frac{1}{V_{\rm h}}\frac{\partial\beta F_{\rm h}}{\partial\rho_i^{\rm h}} = \frac{\partial\beta F_{\rm h}}{\partial(\rho_i^{\rm h}V_{\rm h})} = \frac{\partial\beta F_{\rm h}}{\partial n_i^{\rm h}} = \frac{\delta\beta\mathcal{F}[\boldsymbol{\rho};\boldsymbol{\omega}]}{\delta\rho_i(\boldsymbol{r},\boldsymbol{\omega})}\,. \tag{S6}$$

Defining $\beta\mu_i^{\rm h} \equiv \frac{\partial\beta F_{\rm h}}{\partial n_i^{\rm h}}$, one gets

$$\beta\mu_i^{\rm h} = \frac{\delta\beta\mathcal{F}[\boldsymbol{\rho};\boldsymbol{\omega}]}{\delta\rho_i(\boldsymbol{r},\boldsymbol{\omega})}\,, \quad \forall\,\boldsymbol{r}\in\mathsf{H}_{\boldsymbol{\omega}} \text{ and } \forall\,\boldsymbol{\omega}\in\Omega\,. \tag{S7}$$

Other case studies

Testing and validation: an example with bulk only

In order to prove the technique's ability to calculate the equilibrium condition for totally homogeneous systems, a case from the literature is reproduced: the equilibrium condition of the system for producing synthesis gas from the combustion of methane at 1500 K (T) and

30 atm (P), assumed to behave as an ideal gas[2]. The atomic matrix is

$$\boldsymbol{A} = \begin{bmatrix} 1 & 0 & 1 & 0 & 1 & 0 \\ 4 & 0 & 0 & 2 & 0 & 2 \\ 0 & 2 & 2 & 1 & 1 & 0 \end{bmatrix},$$

where the columns refer to CH_4, O_2, CO_2, H_2O, CO, and H_2, respectively; and the rows to C, H, and O, whose abundances are given by

$$\boldsymbol{b} = \begin{bmatrix} 2 \\ 8 \\ 2 \end{bmatrix} \text{mol} .$$

Although the non-stoichiometric formulation does not require explicit information about the stoichiometry of the associated chemical reactions, it is possible to obtain them from the atomic matrix[2,3]. Thus, the chemical reactions related to the atomic matrix above are

$$2\mathrm{CH_4} + \mathrm{O_2} \rightleftharpoons 2\mathrm{CO} + 4\mathrm{H_2}\,, \tag{S8}$$

$$\mathrm{CH_4} + 2\mathrm{O_2} \rightleftharpoons \mathrm{CO_2} + 2\mathrm{H_2O}\,, \tag{S9}$$

$$2\mathrm{CO} + \mathrm{O_2} \rightleftharpoons 2\mathrm{CO_2}\,. \tag{S10}$$

O'Connell and Haile[2] solved this problem for bulk phase using a stoichiometric formulation of Gibbs energy minimization (GEM), since the temperature and pressure of the system were specified (*bPT ensemble*). In order to reproduce their results, it is necessary to make the *ensembles* compatible and determine beforehand the volume of the system in the equilibrium obtained on those conditions. Therefore, given that the authors reported the solution $\boldsymbol{n}^*$ (Table S1), the corresponding volume can be obtained by

$$V = \frac{RT}{P}\sum_{i=1}^{6} n_i^*\,, \tag{S11}$$

which is approximately 23.68 L, where R is the universal gas constant. Another necessary adaptation is in relation to the reference state. The reference state for the formation properties reported by the authors is that of an ideal gas at a pressure of 1 atm ($P_\circ$). Thus, one obtains

$$\rho_\circ = \frac{P_\circ}{k_B T} \tag{S12}$$

$$\beta\mu_i^\circ = \frac{g_{\mathrm{f}i}^\circ}{RT}, \tag{S13}$$

where $g_{\mathrm{f}i}^\circ$ is the molar Gibbs energy of formation of component i in the standard state, given in Table S1 and taken from O'Connell and Haile[2].

Table S1: Standard Gibbs energies of formation and computed values for equilibrium moles from production of synthesis gas at 1500 K and 30 atm.

i	Species	$g_{\mathrm{f}i}^\circ$ (kJ·mol^{-1})	n_i^* (mol)	
			O'Connell-Haile, 2010	This work
1	CH_4	74.72	0.11420	0.11420
2	O_2	0.00	0.00000	0.00000
3	CO_2	-396.34	0.01885	0.01885
4	H_2O	-164.42	0.09532	0.09535
5	CO	-243.68	1.86700	1.86693
6	H_2	0.00	3.67630	3.67623

The solution obtained via HEM followed the step-by-step shown in Table S2, where $\boldsymbol{\lambda}^0 = [1, 1, 1]^T \cdot 10^{-10}$, $\zeta = 1$ and $\varepsilon = 10^{-8}$. After 37 iterations, the algorithm converged to the same solution reported by O'Connell and Haile[2] with $\boldsymbol{\lambda}^* = [-0.4305674, 1.4750621, -16.8355458]^T$. This example shows that the proposed algorithm expands on the general context of CPE to include a heterogeneous region while retaining the homogeneous CPE as a functioning limit case. Since the purpose of this example is validation only, no attempt was made to improve the initial estimate of $\boldsymbol{\lambda}$ so that the algorithm could converge in fewer iterations. Figure S1 illustrates the path taken by the method with a graph showing the norm of the atomic balance residuals in each iteration. As can be seen, close to the solution, the algorithm shows

a quadratic rate of convergence, as expected from Newton's method.

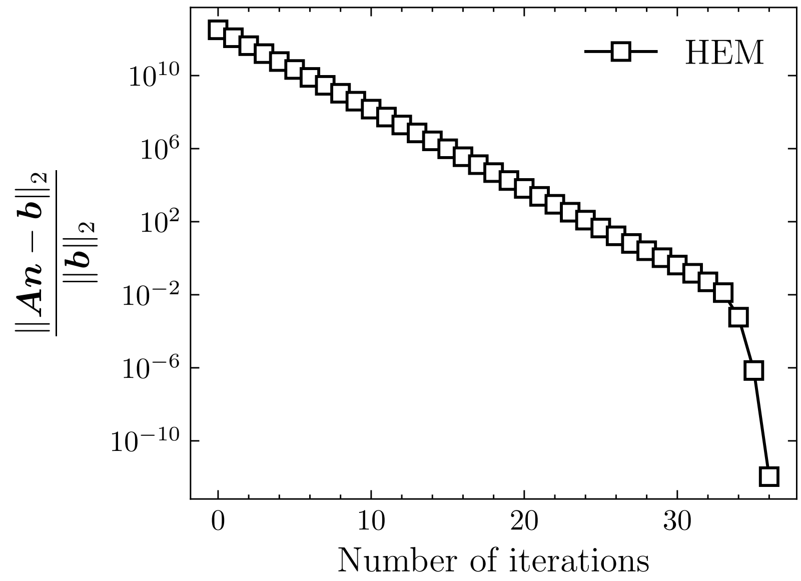

Figure S1: Rate of convergence with HEM in bulk only example. The graph shows the norm of the atomic balance residues in each iteration. In the last few iterations, it can be seen that the rate of convergence changes from linear to quadratic.

An example with multiple reactions and confinement

In this example, we will return to the methane combustion system discussed in the test and validation example. In that section, an equilibrium calculation was reproduced for a homogeneous ideal gas under the conditions proposed in O'Connell. Here, on the other hand, an extension will be made to consider the possibility of the gas being adsorbed in the form of a heterogeneous fluid. It is worth remembering that in this system 3 chemical reactions take place (Eq. S8, S9 and S10), and changes in the system pressure affect the equilibrium composition already for the bulk only case. Similarly to the manuscript I_2 example, consider that the gas is fed into a piston cylinder inside which there is an adsorbent material. The piston allows you to vary the volume (or, if you prefer, the pressure) of the bulk and the question you would like to answer is: how does the conversion of methane vary with the bulk pressure? To answer this, imagine that the temperature and abundances of the gas fed in are the same as in the bulk only example, i.e., T =1500 K and $\boldsymbol{b} = [2, 8, 2]^T$ mol. Assume

Table S2: Step-by-step log of the algorithm for syngas example. The l^2-norm of the atomic balance residual; the l^2-norm of the relative lambda step defined as $\boldsymbol{\delta\lambda}^k = \boldsymbol{\lambda}^{k+1} - \boldsymbol{\lambda}^k$; and the total Helmholtz energy (columns) in each iteration (rows).

k	$\|\boldsymbol{A}\boldsymbol{n}^{\mathrm{b}} - \boldsymbol{b}\|_2^k$	$\|\boldsymbol{\delta\lambda}\|_2^k / \|\boldsymbol{\lambda}\|_2^k$	βF_{b}^k
000	2.723841E+13	6.454920E+09	-1.218141E+13
001	1.002045E+13	9.999862E-01	-8.962583E+12
002	3.686319E+12	4.999778E-01	-4.945725E+12
003	1.356121E+12	3.332896E-01	-2.425907E+12
004	4.988890E+11	2.499064E-01	-1.115552E+12
005	1.835310E+11	1.997907E-01	-4.924663E+11
006	6.751728E+10	1.661874E-01	-2.113629E+11
007	2.483822E+10	1.417537E-01	-8.886409E+10
008	9.137470E+09	1.225295E-01	-3.677768E+10
009	3.361487E+09	1.061070E-01	-1.503306E+10
010	1.236622E+09	9.202134E-02	-6.083388E+09
011	4.549279E+08	8.191008E-02	-2.441404E+09
012	1.673586E+08	7.422099E-02	-9.729875E+08
013	6.156779E+07	6.539321E-02	-3.854761E+08
014	2.264952E+07	5.251692E-02	-1.519380E+08
015	8.332295E+06	3.496103E-02	-5.962122E+07
016	3.065281E+06	4.057100E-02	-2.330431E+07
017	1.127655E+06	7.800046E-02	-9.077530E+06
018	4.148431E+05	9.997537E-02	-3.525019E+06
019	1.526146E+05	1.073881E-01	-1.365096E+06
020	5.614614E+04	1.138240E-01	-5.273611E+05
021	2.065737E+04	1.199996E-01	-2.033032E+05
022	7.601785E+03	1.253247E-01	-7.824881E+04
023	2.798900E+03	1.292470E-01	-3.009447E+04
024	1.032010E+03	1.313206E-01	-1.158777E+04
025	3.819877E+02	1.313824E-01	-4.487172E+03
026	1.428051E+02	1.296588E-01	-1.766012E+03
027	5.467179E+01	1.262752E-01	-7.224320E+02
028	2.188095E+01	1.181710E-01	-3.177878E+02
029	9.068273E+00	9.822005E-02	-1.511982E+02
030	3.585494E+00	8.285744E-02	-7.464335E+01
031	1.269152E+00	7.649627E-02	-4.269368E+01
032	4.311071E-01	4.877285E-02	-3.214176E+01
033	1.112700E-01	1.048319E-02	-2.911825E+01
034	5.019721E-03	3.509586E-04	-2.852468E+01
035	6.037809E-06	4.384050E-07	-2.850334E+01
036	9.305940E-12	6.591466E-13	-2.850331E+01

that the total pore volume is 1.1308 L, that all the pores are slit-like with a width equal to 28.27 Å (H) and that the gas interacts with the solid by means of the Steele potential whose parameters are given in Table S3. The Figure S2 shows the conversion of methane

Table S3: Steele potential parameters for methane combustion species.

i	Species	ϵ_i [K]	σ_i [Å]	$g^\circ_{\mathrm{f}i}$ (kJ·mol^{-1})
1	CH_4	1982.63	3.579	74.72
2	O_2	1546.20	3.4335	0.00
3	CO_2	2390.01	3.6705	-396.34
4	H_2O	3295.10	3.0205	-164.42
5	CO	1528.01	3.545	-243.68
6	H_2	951.03	3.1135	0.00

as a function of bulk pressure. The dashed black curve indicates the methane conversion if the system were bulk only; the purple curve indicates the global methane conversion, i.e. throughout the system (considering both bulk and pores); and the green and orange curves indicate the methane "conversions" in the pores and bulk respectively. The fact that the word is in quotation marks is due to the fact that these conversions do not actually measure exactly how much methane has been consumed by the chemical reactions in these regions, but rather how much methane has disappeared from each region, regardless of whether due to chemical reactions or adsorption/desorption processes. Thus, at the point where the orange curve reaches 100%, there is no more methane in the bulk, either because it has been completely consumed or adsorbed. And that's why the sum of these two curves doesn't result in the purple curve. Instead, it holds that $X_i^{\mathrm{b}} + X_i^{\mathrm{h}} = X_i + 1$, where X_i^{b}, X_i^{h} and X_i are, respectively, the conversions in the bulk, in the pores and in the system. In any case, the difference between the curve and the dashed black line is the contribution of confinement to conversion. This effect is more pronounced at high pressures, since it is under these conditions that adsorption is most favored. As can be seen, adsorption tends to reduce overall conversion. At low pressures, the purple and orange curves converge towards the limiting behavior represented by the dashed black curve. Under these conditions, adsorption

is disadvantaged and the system is dominated by the bulk. Thus, global and bulk conversions are approximately the same. The "conversion" of adsorbed methane tends to 100% because its adsorption does not occur in a relevant magnitude.

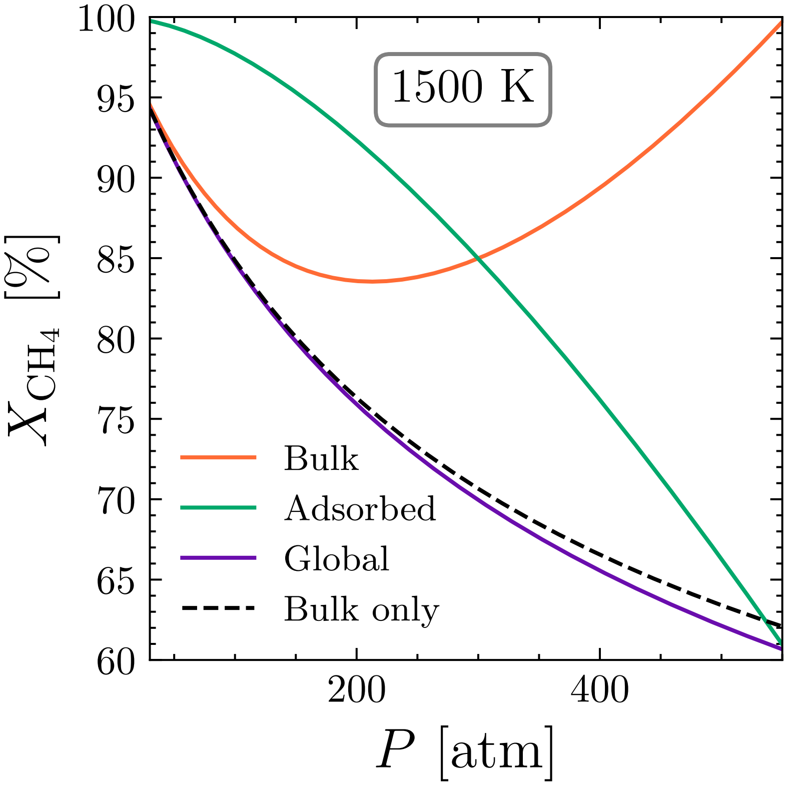

Figure S2: Conversion of methane as a function of bulk pressure in combustion system. The dashed black curve indicates the methane conversion if the system were bulk only; the purple curve indicates the global methane conversion, i.e. throughout the system (considering both bulk and pore); and the green and orange curves indicate the methane "conversions" in the pores and bulk respectively.

References

(1) Engel, E.; Dreizler, R. M. *Density Functional Theory - An Advanced Course*, 1st ed.; Springer Berlin, 2013.

(2) O'Connell, J. P.; Haile, J. M. *Thermodynamics - Fundamentals for Applications*; Cambridge University Press: New York, 2005.

(3) Smith, W. R.; Missen, R. W. *Chemical Reaction Equilibrium Analysis: Theory and algorithms*, 1st ed.; Wiley, 1983.
